# Developing professional ethical guidance for healthcare AI use (PEG-AI): an attitudinal survey pilot

**DOI:** 10.1007/s00146-025-02276-z

**Published:** 2025-03-12

**Authors:** Helen Smith, Jonathan Ives

**Affiliations:** 1 Centre for Ethics in Medicine, Bristol Medical School, University of Bristol, Canynge Hall, 39 Whatley Road, Clifton, Bristol BS8 2PS, England

**Keywords:** Artificial Intelligence, Healthcare, Ethics, Professional guidance, Qualitative research

## Abstract

Healthcare professionals currently lack guidance for their use of AI. This means they currently lack clear counsel to aid their navigation of the problematic novel issues that will arise from their use of these systems. This pilot study gathered and analysed cross-sectional attitudinal and qualitative data to address the question: *what should be in professional ethical guidance (PEG) to support healthcare practitioners in their use of AI?* Our survey asked respondents (*n* = 42) to review 6 themes and 15 items of guidance content for our proposed PEG-AI. The attitudinal data are presented as simple numerical analysis and the accompanying qualitative data were subjected to conventional content analysis; the findings of which are presented in this report. The study data allowed us to identify further items that could be added to the PEG-AI and to test the survey instrument for content and face validity prior to wider deployment. Subject to further funding, we plan to take this work further to a wider study involving the next iteration of this survey, interviews with interested parties regarding PEG-AI, and an iterative Delphi process (comprising an initial co-creation workshop followed by iterative consensus building) to enable experts to reach consensus regarding recommendations for the content of PEG for AI use in healthcare. We aim for this work to inform the healthcare regulators as they develop regulatory strategies in this area.

## 1 Introduction

The United Kingdom (UK) Government is investing heavily in the development and deployment of artificial intelligence (AI) in healthcare (Department for Science, Innovation and Technology, Department of Health and Social Care, 2023). The drafting of guidance tools related to AI in healthcare have historically been directed towards its creators and those who would purchase it (NHSX 2020; Central Digital & Data Office, 2020; Department of Health and Social Care, 2021); however, little attention has been paid to the guidance needs of those healthcare actors who will be using AI.

AI adoption introduces non-human actors into the multidisciplinary team at patient bedsides (Smith et al., 2023a, raising not just practical, but also the ethical and professional questions about how it can and should be used. These questions must be asked and answered prior to widespread AI introduction to ensure safe, appropriate, and equitable AI use in healthcare.

We cannot look to AI-specific legislation to equip us for this future. Instead of a core cross-sector regulatory framework which stipulates the standards to be achieved in AI use regardless of its application (e.g. as is happening in other jurisdictions such as Europe’s 2024 Artificial Intelligence Act), the UK is taking the stance that specific regulators should “provide effective rules for the use of AI within their remits” (Secretary of State for Science, Innovation and Technology, 2024). For example, in England, all registered HCPs are regulated by their respective regulatory bodies, such as the General Medical Council (GMC, 2024), the Nursing and Midwifery Council (NMC 2018), and the Healthcare Professions Council (HCPC, 2016). These set out professional codes of conduct to stipulate the behaviours expected from registrants when practicing. However, these professional codes of conduct do not mention the use of AI, and given this it is perhaps surprising that NHS AI Lab & Health Education England (2022, p.34) state that “clinicians look to regulators for guidance on how they should use AI technologies and for reassurance that using AI in clinical practice will not threaten their professional registration”. There is clearly a mismatch, here, between expectation and provision, meaning that healthcare professionals are faced with using technologies without clear guidance to help them navigate problematic novel issues related to these systems.

Professional guidance is not entirely lacking. The trade union and UK professional body for the diagnostic imaging and radiotherapy workforce, the Society of Radiographers (2021), for example, have generated their own baseline guidance for AI-enabled radiography practice. This is excellent to see, however, none of the healthcare professional regulators have followed suit yet and, if they did, it would be an unmanageably fragmented approach if each professional regulator or specialism produced their own guidance. The proliferation of multiple sets of standards (Munroe, undated), risks inconsistent approaches across the healthcare professions (Smith et al., 2023aa), creating the potential for disharmonious working practices in multi-disciplinary healthcare settings. We recognise the need for harmony in guidance (World Health Organization 2021) to promote equity in practice across all healthcare sectors, and have called elsewhere for unified inter-professional/specialism guidance for AI use to be developed for all healthcare professions (Smith et al., 2023a).

Unified inter-professional/specialism ethical guidance for AI use (PEG-AI) could undergird any future profession/specialism-specific guidance. PEG-AI will reduce the risk of potential AI-related harms by highlighting to HCPs the foreseeable nature of those risks and encourage reflection and action. For example, PEG-AI could help HCPs identify and mitigate AI-related risks by guiding them to perform critical evaluation of all AI recommendations prior to using them, thereby mitigating ‘atrophy of vigilance’ (Ruskin et al. 2020; Smith et al., 2023a).

Our pilot work asked the research question: *what should be in professional ethical guidance (PEG) to support healthcare practitioners in their use of AI?*

The work undertaken in this exploratory pilot project is an exercise in ethical foresight analysis (Floridi and Strait 2020) where the ethical implications of AI use are identified to inform recommendations that underpin the development of PEG-AI use in all healthcare sectors. This anticipatory approach is preferable to organisations reactively and separately determining standards after preventable harms have affected patient wellbeing, which would have a negative reputational effect upon both the healthcare professions and AI adoption (Smith et al. 2023a). This kind of work is best done in consultation with affected persons, as opposed to being undertaken by individual teams of researchers relying solely on their own experiences and perspectives. The inclusion of groups from all corners of society enables members to not only express their concerns, but also be part of developing, shaping, and deploying the solutions to issues identified during research activities. Without participation, researchers risk missing the challenges that marginalised groups face, thereby alienating them further.

This project sought to develop and pilot a survey to engage relevant affected persons in thinking about the scope and content of PEG-AI, with a view to informing the development of wider-scale consultation and guidance design. We aimed to both capture perspectives about what should be included in professional ethical guidance (PEG) to support healthcare practitioners in their use of AI, and to assess our survey tool for face and content validity.

## 2 Methods

A cross-sectional survey was designed to gather attitudinal data from participants regarding their views about the contents of PEG-AI in healthcare. Broadly, we wanted to hear from:

- Healthcare professionals registered with one (or more) of the 10 regulators overseen by the Professional Standards Authority for Health and Social Care (2024).
- Working for one of the 10 regulators overseen by the Professional Standards Authority for Health and Social Care
- Academics
- Patients
- Interested members of the public

Whilst the above appears restrictive to just five groups, in practice it was very inclusive. All people have bodies which will require them to access healthcare at some point in their lives; ergo, we considered *all* people to be affected parties in this topic. Inclusivity was central to this study. Reflecting this, the call for participation specifically encouraged inclusion of people from diverse groups, such as those with protected characteristics as per the Equality Act 2010 and other characteristics that the Act does not cover, e.g., socio-economic disadvantage. We considered the recruitment of diverse voices essential to this study, as disadvantaged/minority groups have already come to harm through the deployment of AI (Obermeyer et al 2019, Sjoding et al. 2020, Trendall 2019). Any PEG-AI developed ought to be informed by these groups to reduce the risk of future practices harming them further.

To further support this aim, when asking about their demographics we invited participants to self-describe their characteristics of gender, ethnicity, and sexual orientation (rather than using standard categories). We did this as we wished to avoid participants from having to tick a box that described their characteristics as “Other”; ‘othering’ participants risks inserting a barrier to their equitable engagement with our research activities (Smith et al. 2022).

Recruitment took place via social media advertising and contacting existing networks of people interested in AI in healthcare. We additionally invited participants to suggest groups/organisations whose members might be interested in responding to the survey, especially those at risk of experiencing inequality arising from AI use. Any groups/organisations suggested by participants were subsequently approached, where we invited them to share the link to the survey within their networks.

As this was an exploratory pilot, we did not aim to test a hypothesis or explore cause or correlation. As such, a power calculation was not required, and we simply recruited as many respondents as we could within the limited **4**-week period the survey could be kept open for. This report offers descriptive statistics, reporting frequencies of responses—which gives insight into what participants viewed as ethically important (and why) and what should be covered in PEG-AI, without any claim to be representative of generalisable.

Our survey presented participants with a variety of ethical themes and guidance content that might be included in PEG-AI, inspired by:

- The Society of Radiographers (2021) AI working group’s guidance for clinical imaging and therapeutic radiography professionals
- The Department of Health and Social Care’s guidance for digital and data-driven health technologies
- WHO’s (2021) Ethics and governance of artificial intelligence for health
- And UNESCO’s Recommendation on the Ethics of Artificial Intelligence

Multiple choice questions using a 5-point ordinal scale (Sullivan & Artino 2013) were posed, allowing respondents to tell us how important each theme and piece of guidance content was to them (not important, somewhat important, important, essential, or don’t know). We chose not to ask participants to rank the importance of each of the themes or guidance content as we were concerned with absolute, as opposed to relative, importance.

For each item, we offered an optional free text response enquiring why a score had been given and what, if anything, they would change about the item. Additionally, we asked participants what other content should be added to the PEG-AI, and which actors should be held responsible for patient harms due to AI use in healthcare.

The survey was developed by HS, with contributions and initial review by JI. It was then reviewed for face and content validity by two experts in AI and ethics research (Kenton O’Hara and Matimba Swana). Their feedback led to changes and simplifications to the content and style of the survey. It was then further reviewed by (Giles Birchley), who offered further feedback but did not suggest any changes to the instrument itself. This process established initial face and content validity.

Once gathered, we explored the data for signs that the survey lacked content or face validity, to help us further refine the survey instrument prior to deployment in a future larger study. We wanted to know whether the questions sensible/appropriate/relevant, and whether they covered what we intended them to cover? (Connell et al 2018). This involved us exploring the data for participant responses that indicated whether the survey had asked confusing or nonsensical questions, and looking for (in)consistency between answers to the ordinal scale and open text questions. We additionally asked respondents for feedback about the quality of the survey. This allowed us to identify areas of unclarity or lack of relevance.

### 2.1 Data collection, participants and sampling

The survey was constructed in Microsoft Forms, hosted on the University of Bristol’s secure servers, and was open from 14th May to 14th June 2024. The survey presented 76 questions to all participants, with a further question asked of each HCP or regulator participant to determine which regulator they were registered with or worked for. The participants who were otherwise interested persons were also asked an additional question inviting them to describe their interest in PEG-AI. Unforeseeably, the UK General Election was declared on the 22nd May. We were told by one potential respondent that they were not allowed to participate in this survey as they had to remain politically impartial during the pre-election period due to their occupation. As such, we recognise that the pre-election period may have influenced recruitment to this study.

## 3 Analysis

The quantitative data responses to the 5-point ordinal scale for each of the themes and guidance content are presented in the results section of this paper in Tables 1 and 2. We performed a simple descriptive numerical analysis upon these data, to give a quantitative indication of participants’ overall views about the PEG-AI presented to them.

Qualitative data collected in response to open questions were analysed using conventional content analysis involving “the subjective interpretation of the content of text data through the systematic classification process of coding and identifying themes or patterns” (Hsieh & Shannon 2005). We focused on any reasons and/or explanations that were given for the significance of any particular item. The analysis comprised open coding the raw data (in grounded fashion), followed by collating codes into categories that described and captured content. All qualitative data were read and allocated initial codes. The codes were then sorted into categories that captured reasons and explanations linked to specific proposed PEG-AI themes and guidance content. All categories, subcategories, and codes were clearly defined in a code book (Hsieh & Shannon 2005).

HS performed the primary data analysis, with JI checking and double coding around a quarter of the data (10 out of the 42 responses).

## 4 Ethics

This study was approved by the University of Bristol’s Faculty of Health Sciences Research Ethics Committee (FREC) Ref: 18,362.

## 5 Results

We are very grateful to the 42 people who responded to this survey. A brief overview of the respondent demographics is offered in Table 3.

Whilst this project had aimed to recruit people from a broad cross-section of society, we found that patients and HCPs were the two largest participant groups. Additionally, across all participant groups, we attracted an over-representation of disabled people (*n* = 18), around twice that of the UK population’s average of 24% (UK Parliament 2023). Responses from other groups, e.g., practicing legal professionals, would have been highly welcomed and greatly informative, but, given that patients, HCPs, and disabled people will be directly affected by the adoption of AI in healthcare, it felt fitting that we received the greatest engagement from these groups. We noted over-representation from those in the gender binary who described themselves as female, those who predominately described their ethnicity as White, and those who described themselves as heterosexual. Comprehensive and diverse representation is impossible with such a small sample size, but these data underline the importance of promoting further inclusive engagement from minoritised groups in later iterations of this work. We recognised that inequality is not constrained solely to those characteristics defined by the Equality Act 2010 and so invited participants to share with us a description of characteristics that they have which are not covered by the Equality Act. We can’t speak to the representation of those whose characteristics were not protected by the Equality Act as there are no national statistics with which to compare, but we were grateful for participants sharing these particulars with us so that we had an opportunity to further appreciate their personal contexts. ^1^

The survey asked participants to review 6 proposed themes and 15 items of proposed guidance content, and we offer the quantitative data in Table 2 and a high-level report of our qualitative analysis below. Our respondents gave generously of their thoughts and ideas; we have focussed our reporting to that which directly addresses the ethical issues of AI being used by HCPs. In the reporting that follows, quotes were chosen from the data that best illustrate and evidence the key points raised by participants in response to the questions asked. Numbers in curly brackets after quotes indicate the corresponding participant numbers.

### 5.1 Guidance themes

Table 2 shows that broadly, participants found the six PEG-AI themes we suggested to be agreeable.

All the guidance themes proposed were considered ‘essential’ or ‘important’ by vast majority of respondents, but some more so than others.

#### 5.1.1 Preventing patient harm from arising due to AI use

Preventing patient harm garnered greatest agreement; respondents felt that AI adoption must not lower professional standards and that patient-centred care must remain the priority:

“The whole point of healthcare is to improve patient wellbeing and health, not to harm it, therefore this is fundamentally important” {33}.

AI was felt to have the potential for both benefit and harm, with ‘communication’ offered as an example of an area where AI could bring clarity or create more problems than already exist. Some respondents raised concerns that HCPs might not recognise how using AI may cause harm:

“…current approvals are based on systems being used to assist clinicians make decisions within their own level of competence, but since there is no way to control this, it is likely AI systems will be used by less experienced users who may act outside their level of competence. Such users are unlikely to be aware of the limitations of such systems” {29}.

This implied a need for HCPs to be educated prior to AI adoption, for example, by knowing the limitations of an AI prior to using it, to ensure it does not cause harm.

#### 5.1.2 Ensuring fairness, inclusiveness and equity

Respondents recognised these as core principles to good clinical practice. Fairness was described as essential, with good equality and diversity reported to be needed for all healthcare:

“Good equality and diversity practices make sure that the services provided to people are fair and accessible to everyone and everyone is treated with dignity and respect.”{40}

Issues with inequality were acknowledged to be already present and participants did not want AI to exacerbate this:

“It would be unacceptable for the NHS to bring in a technology that worsens the inexcusable health inequity we have within our society.”{30}

The participants raised concerns about the diversity and inclusiveness of data encoded into AI. For example, bias in the data used to train AI in dermatology:

“I’m a dermatologist and much of our AI is not fit for skin of colour (currently mostly for skin cancer detection) so this raises significant concerns”{16}

#### 5.1.3 Protecting and enhancing patient autonomy

Respondents described respect for patient autonomy as being core to healthcare. They noted that whilst AI could empower people and patients, for example, by offering more information to patients, not all informational needs will be the same. Different patients would have different informational needs and AI would need to be responsive to this.

“Most often during my clinical practice patients want me as their healthcare professional to make decisions on their behalf based on my professional judgement with clear communication and opportunity to ask questions where needed. I think a smaller proportion of people want to have full autonomy over their health within our increasingly complex healthcare environment.”{30}.

Broadly speaking, the respondents were concerned with how patient autonomy could be affected if AI widened the gap between patients and HCPs. This was described in terms of communication and the importance of human participation in decision making.

“As someone with a chronic illness, I know how important it is to my wellbeing to feel involved in my care. I don’t want a computer to make decisions for me. Even if an AI model could calculate what treatment path is likely to give the best outcomes, it would never be able to consider my individual circumstances, opinions and values, and I would still need to be involved in decision about my care.”{11}.

Some participants stressed the importance of patient choice about whether AI systems are used in their care, but another pointed out that:

“AI systems are being widely deployed in healthcare systems with no input from patient groups. Most will be unaware such systems were used in their care.”{29}

This point might reasonably lead us to wonder whether this status quo is already threatening patient autonomy, or perhaps it shows that worries about future AI use eroding autonomy are unjustified.

#### 5.1.4 Protecting and enhancing HCPs’ autonomy

Respondents recognised the benefits of AI to HCPs, but tended to feel that that HCPs should treat AI as a tool that supports, rather than dictates to, them:

“HCP’s need to know that they are making what they feel is the best course of treatment—AI can back this up but must not dictate”{14}

Some cautioned that there need to be limits to HCP autonomy and that they must operate within guidelines. Others raised concerns that AI will insult HCP autonomy, confidence, and freedom in their own decision-making:

“Medicine is nuanced, unpredictable and variable and I would be concerned if HCPs’ confidence in their own professional judgement was impacted, but I would also be concerned that the freedom (from a regulatory perspective) for those HCPs to make that professional judgement may be impacted too”{30}.

Concerns about limits to professional freedom in AIrelated decision-making tended to be future oriented. There was a feeling that HCP autonomy is currently protected by their ability to ignore an AI, but they anticipate that in the future HCPs will be constrained to follow AI and professional autonomy will be eroded.

#### 5.1.5 Accountability: (a HCP being able to fully justify their decision to use an AI)

Respondents were very clear about HCPs needing to be accountable for AI use:

“If HCPs have autonomy over the choice to use AI, it is essential that they can justify their decision to use it.”{37}

Respondents valued transparency and explainability in AI design and use and the importance of audit:

“…at a doctors appointment I not only expect my doctor to know exactly why decisions are being made, but I expect to be able to ask questions and have them answered. Not only this, but if something goes wrong with my treatment I want to know that there’s a paper trail documenting the reasoning behind each decision, and that each professional involved is able to answer questions in depth about what went wrong. If AI has been involved it would need the same transparency.”{11}.

They noted that principles of clinical governance would be needed for AI use, especially if an HCP delegates decision-making to the AI. They also felt that HCPs would need information about and from their AIs so that their choice to use the AI could be accountable. Further, it was noted that, to be accountable, HCPs will need training in AI-related practice.

#### 5.1.6 Responsibility: (a HCP can be praised or blamed for the outcome of their use of AI)

The respondents agreed with this theme and felt that HCPs ought to be responsible for recognising the foreseeable consequences of their using AI. However, they cautioned that HCPs might not be aware that responsibility for AI use remains with them as users and not the organisation that developed it.

Respondents also noted that responsibility allocation is not simple, and raised a variety of discussion points. To start with, the allocation of responsibility was identified as an issue related to procurement as it’s not necessarily HCPs that make AI adoption decisions:

“The responsibility should lie with the hospital/trust and there should be strict and robust procurement procedures and regulation in place before widespread use.”{30}

But it is not just who chooses to adopt the AI, the proximity between the HCP and the AI is also key to responsibility allocation:

“…when an AI system is acting completely independently—almost like another HCP—then it would be unfair to praise or blame the HCP for the outcomes.”{37}

One respondent suggested looking forward after incidents: that insight, change, and making amends is more important than allocating blame for harms.

“I feel like blame is less important than insight and then change, as well as compensation where applicable.”{11}

Finally, the respondents indicated that they wanted concrete guidelines for responsible AI use: they told us that responsibility allocation needs to be determined so that safeguards for AI use can be developed; e.g., regulation and regulatory guidance for AI use is needed for HCPs.

### 5.2 Guidance content

Respondents generally reported the PEG-AI guidance content, we suggested to be acceptable (Table 2), with answers to open text questions to explain why they felt certain content was more or less important.

#### 5.2.1 An AI can only be used if it has been approved by an authoritative body (such as The National Institute for Health and Care Excellence)^2^

Participants recognised that AI should be evaluated and approved just like any other healthcare technology:

“AI tools in healthcare should be objectively evaluated and validated in the same way as other devices, medications and technologies”{18}

The reasoning for this was simple (and consistent with the principle of ‘preventing harm’): safety in healthcare is paramount.

It was recognised that HCPs cannot personally assess every AI deployed and a topical example was offered:

“[we must know that] patient data is kept within GDPR, if using chatbots, how can you be sure the software will respect patient consent?”{33}

One respondent articulated the risk that clinicians might assume that if an AI is in routine use then it must have been properly developed and tested, which might not be the case. We know that HCPs typically examine the research evidence-base to inform their practices (Connor et al 2023), but one respondent expressed concern that healthcare assessments made by AI will not live up to traditional evidence-based methods of practice. Respondents’ feeling that AI should only be deployed after approval from an appropriate authority seems to recognise that AI is complex and that HCPs are unlikely to be in a position to evaluate its performance or outputs.

Some respondents noted that review of AI by experts is important, but that gaining approval for technology can be a barrier to adoption because approvals take time. However, the responses elsewhere told us that preventing harm and maintaining safety in healthcare is a priority and we see this point about approvals creating barriers to be highlighting a problem with process, rather than suggesting speed should be prioritised over safety.

#### 5.2.2 HCPs will only use the AI for purpose it has been designed

Respondents reported split views about this. Some felt that to only use AI for the purpose it was designed might stifle innovation—is there an off-label approval that could be developed? One respondent noted that there is a history of HCPs using tools for different uses:

“e.g., A HCP can sometimes be creative with medicine prescriptions—e.g., prescribing diabetes medications for weight loss.”{37}

Respondents noted that there could be reasons to use an AI for purposes other than that for which it was originally designed, and that there might be good reasons to build flexibility into PEG-AI use—albeit with appropriate safe-guards such as requiring ethical approval, as one participant suggested.

Conversely, other respondents defended a more cautious approach, suggesting that off-label use risks harm and therefore should not be practised:

“Very important to use as designed as this will be most likely to have been evaluated and been deemed safe to use in this way”{27}

The priority that respondents tended to give to safety, combined with their general scepticism about HCPs ability to really understand the technology, suggests that off label use is considered risky—which was accepted even by those who supported it. This suggests that any flexibility with regard to off-label use could only be supported with appropriate safeguard.

It was also noted that users of AI cannot assume that any given technology will remain safe and appropriate to its use case. Training data, configuration, or environment will change over time, meaning that safety assessment must be a continuous process—pointing to the need for an item referring to this in PEG-AI:

“The performance of AI is highly sensitive to changes in the data landscape and HCPs will not have the tools or expertise to assess whether this variable performance is within safe parameters.”{30}.

#### 5.2.3 HCPs will follow information governance and data protection guidance

Respondents tended to view this as a “core principle of healthcare”{1}, with AI being no exception to the need for data regulation compliance. It was felt to be ‘tedious’, but important:

“although tedious to follow, processes do force minimum standards of behaviour, prompt important considerations, and provide audit trails for future learning”{24}

The importance of data protection was framed in terms of privacy and preventing misuse of data, which were linked to trust:

“It’s impossible to build trust in AI without ensuring patients’ data is safe.”{11}

It was also suggested that data governance needs to be frequently reviewed to stay up to date with advancements in AI.

#### 5.2.4 HCPs will mitigate for and report AI issues appropriately (e.g. Medicines and Healthcare products Regulatory Agency’s Yellow Card Scheme) when they arise (for example, suspected error, suspected bias etc.)

Participants agreed with this item suggestion and saw AI as a tool like any other, and faulty tools need to be reported. The analysis of feedback via a transparent reporting system was felt to be needed, so that issues could be detected and lessons learned, in order to promote safety.

It was suggested that reporting needs to be non-onerous to be used by HCPs: ”HCPs need to be aware of reporting responsibilities and the process for reporting needs to be made simple and easy to access so that HCPs who are under immense time and resource pressure can feasibly report adverse events.” {30}

But, to do this, HCPs need to be able to spot AI errors, and this will require HCPs to be knowledgeable about how and when to report issues. To be in a position to do this, it was noted, HCPs would need to maintain their clinical knowledge and skillset, and retain an oversight role:

“HCP’s should still have the skills to follow best practice, personal experience and gut feeling, knowing their patient and their wishes.”{19}

#### 5.2.5 AIs will be taken out of service if it is suspected their use will result in unmitigable risk of harm/injury

This was considered by many participants to be so ‘obvious’ as to not need explaining, although some gave specific examples, for instance:

“Very important that AI use does not exacerbate already significant health inequalities e.g. diagnosing skin lesions in people of colour”{18}

A low threshold was suggested for withdrawing AI if concerns are raised:

“just being suspected is enough to immobilise the AI” {4}.

The need for a reporting timeline with dedicated monitoring/tracking for AI was mooted. It was, however, also noted that a balanced approach is needed to prevent an otherwise beneficial system being taken out of service:

“…every drug we use has the potential for harm / side effects some of which can’t be mitigate[ed]. AI should [be] treated in [the] same way rather than setting a much higher bar and shutting down systems that may have many benefits”{22}.

#### 5.2.6 HCPs will not knowingly use an AI that is biased against the patient’s characteristics unless they are confident that they can mitigate the use of any output given by the AI

Respondents tended to feel that an AI ought not to be biased prior to deployment to ensure safety and ensure that inequalities are not perpetuated and this might suggest this guidance content is not needed.

But the issues were complex, with some respondents noting that a prior step needs to be eliminating implicit and explicit bias in the healthcare system in general. It was also felt by some that, there is no such thing as an unbiased AI and that bias can work in favour of disadvantaged groups:

“Some AI can be bias in favour of disadvantaged populations and in this case the AI is negatively bias against those not already experiencing worse outcomes. I also think it is unachievable for AI tools to be totally unbiased— the nature of these tools is that they display disparate performance as a result of variable training/testing data. We cannot remove the imperfections and influence of biased health data.”{30}.

It was further noted that, given there is already bias in the current system, we ought to be open to the possibility of AI being less biased than the status quo:

“depends-[if it is] better than the status quo then might still be benefit in using even if not perfect”{22}

#### 5.2.7 HCPs will challenge, mitigate, or reject an AI output if it is unfair to groups or individuals

The respondents agreed that users must retain decision-making autonomy and balance the benefits and risks of AI use. Ideally, an AI will be perfected prior to adoption so that (already pressured) HCPs should not have to compensate for AI deficiencies.

“It is important to challenge output that appears discriminatory so that these issues can be rectified, ideally before an AI interacts with a patient population. Otherwise, this puts patient at risk. I do not believe however that HCPs should have to routinely compensate for biased AI models-these should be amended at a technological level (eg by addressing biased training data).”{11}.

But some respondents also asked how HCPs would be able to recognise problems with AI and challenge it. Further they asked whether HCPs would have the authority to challenge an AI, and wondered whether they would have to notify patients as and when it happened? There was also a suggestion that HCPs would need clear processes and an independent body to support them if they needed to challenge AI.

#### 5.2.8 Patients should know when AI is used in clinical practice

Respondent views were mixed on this item, with some feeling that patients need to know, and others feeling they did not need to be specifically told.

Those who felt patients should be told about AI use tended to report that transparency and honesty are crucial and that this required patients having the option of knowing if AI was planned to be used in their care. This was felt to be essential in respecting autonomy, i.e., by respecting patients’ right to opt out of AI use in their care:

“this is critical to patient autonomy and choice, accountability and ethical use of AI in healthcare. I want to know when AI is a part of my care. I want the choice to opt in or out. I want to know how my data is used. I want to ask question[s] about how the AI and my HCP made decisions about my care, especially if something goes wrong. I want to [know] who made the AI, whether they’ve had any issues with patient safety or data breaches, what other patients say. I can’t know any of this if I’m not made aware that AI was involved in the first place.”{11}.

The contrary view expressed suggested that the need to inform patients about AI use would depend on how it is used:

“If the HCP is in charge a the AI system is merely a tool, I don’t think patient’s always need to know, as [they] don’t know about every other tool that is used in clinical practice.”{37}.

The idea here seems to be that HCPs do not routinely explain and identify every tool they will be using, there is no reason to think that AI, used as a tool, should be any different. There was one suggestion that there should be a blanket statement/policy that AI is used in healthcare, meaning that individuals need not be told separately.

#### 5.2.9 Prior to AI use HCPs will gain informed consent from those whom the AI recommendation will affect

Two broad views were articulated here, broadly tracking the views described above. One view holds that express consent for AI use is not needed and that consent would only be needed if AI recommendations are enacted without human oversight—for the reason outline above.

The other holds that express consent for AI use is needed as it ensures patients can be informed:

“too often, consent is just ar*se-covering [sic], but it would at least give patients a chance to ask questions about the AI if they hadn’t thought about it before”{24}

However, the above assumes that patients will actually be in a position to be informed and make a choice about the involvement if AI in their care and one respondent mused over whether this would be real choice if there were no meaningful alternatives:

“What options do the patients really have if they don’t want AI. Is this just passing the buck and blaming the patient for agreeing to something they have little chance of really understanding and no option but to agree to. If they say no, then what? No treatment?”{20}.

#### 5.2.10 Patients with capacity can reject AI use in their care

Many respondents felt that patient autonomy should be respected by allowing them to reject the use of AI in their care—even if it was considered and unwise decision:

“Patients bring their own biases, and (like those opposed to vaccines), might inadvertently make their care worse.”{29}

We were told that people’s trust in healthcare services might be affected if they are unable to refuse AI use in their care. However, one participant was concerned about consent being used to shift responsibility for AI use from HCPs onto their patients.

Consistent with what is described above, several respondents wondered whether it will it be possible to reject AI use in the future and what the implied for whether there would be a meaningful choice:

“If AI use where to become standard practice or the ‘gold standard’ and non-AI use was inferior or not available would they be able to reject? E.g. if AI scan reporting became normal and humans became deskilled or not available to ‘manually report’”{18}. “Patients should have the right to say no but there should also be a suitable equally available option for treatment if they do otherwise it’s not really a right that can be enacted.”{28}.

#### 5.2.11 A HCP will not relinquish their role in patient care to an AI. They will determine if/when it is appropriate to use, and use their clinical knowledge to justify accepting or rejecting the use of an AI recommendation

This item also split our respondents. Some of those who rejected it wondered what the point of AI is if the HCP continues to make all decisions and carry all responsibility themselves.

These who accepted this item felt that HCPs should retain oversight of the AI because AI is not perfect, and that HCPs retaining their role is important to promote safety, to individualise care to each patient and to monitor and intervene as needed where AI is used:

“Part of the role of being a professional is to act as a “learned intermediary”, not just accepting any and all advice you receive.”{26}

#### 5.2.12 HCPs will only use AIs when practicing within their competence; AI is not a substitute for a knowledgeable and experienced HCP

Many respondents noted that human oversight is essential as AI can be unreliable:

“human oversight and opportunity to intervene must be retained”{24}

They stressed that AI should not be the single factor guiding decision-making and that HCPs should draw on their own knowledge and experience; ergo, AI should assist HCPs rather than replace them.

It is worth noting that some respondents seemed to work on the assumption that AI will always be imperfect and require oversight for safety, whereas others seemed to assume that there might be point at which AI becomes as safe, if not safer than human HCPs. As such, it might be that the relevance of this item, and others above, is dependent on the state of the technology.

#### 5.2.13 HCPs will be able to account for/justify their use of an AI recommendation

Participants saw accountability as part of a HCP’s compliance with their professional regulation, and that being able to account for AI use was essential for safety. However, they acknowledged that user accountability will be limited by HCPs’ understanding of AI:

“it is not reasonable to expect HCPs to understand fully how an AI works”{24}

#### 5.2.14 HCPs will keep adequate record keeping regarding their AI use

Respondents saw record keeping as part of HCPs’ professional duties; important not only for transparency and accountability, but also for the purpose of auditing both patient care (when determining what does/not work well for that person, and in case of future litigation) and AI performance:

“Record keeping is always essential both for deciding future treatment and checking back if things go wrong.”{13}

#### 5.2.15 HCPs will know that they are responsible for their use of AI and the effects of that use

Whilst respondents noted that humans, not AI, ought to be responsible for the consequences of AI use their views about this item were not entirely clear-cut.

Being responsible for one’s actions was reported to be part of being a HCP and there was broad agreement that HCPs ought to be responsible if they used AI themselves. One participant, however, asserted that HCPs should hold joint responsibility with the employing organisation that commissioned the AI.

It was suggested that HCPS have a duty of care and being held responsible for the consequences of AI will promote conscientious use:

“this will motivate [HCPs] to engage with the product and understand what it does”{24}

The importance of education was once again noted, so that HCPs are aware of responsibility they will bear when using AI.

One respondent noted that HCPs need a viable alternative option to using AI, or else they become responsible for the choices of others—drawing on idea that we can only be responsible for what we have control over:

“This only works if HCPs have a viable option to not use it or to use a better alternative. Those on high who have never seen a patient implement things like this and then make us ‘responsible’ for everything that then happens. We already know we are responsible for absolutely everything that happens to the patient whether it is actually our responsibility or not.”{20}.

### 5.3 What did we miss?

Whilst we were reassured by our participants’ positive reception to the themes and guidance content set out in this pilot work, we were not convinced that we had captured every possible item that ought to be considered. Our participants allowed us to compensate for the risk of missing items by suggesting them to us.

The respondents had several suggestions for items that should be included, but these mostly revolved around factors prior to implementation (e.g., that AI should be carefully designed and be cost effective), and so were out of scope. There was, however, a recurring theme around HCPs’ knowledge, to which there were three parts.

One was that HCPs will need training prior to their using AI to ensure that it could be used safely:

“HPC’s are accountable for their decisions and the HPC will have to deal with any improper use and professional errors, as the HPC has done since its beginning. HPC’s can also be struck off, but who will then repair the damage? They will need proper education as part of their HPC registration and [continuing professional development] follow up if AI is being used for our treatment”{12}.

The second was that HCPs need to retain clinical knowledge and skills, even when relying on AI, so that they can practice without AI when needed (including, for example, if AI fails):

“I think AI could revolutionise some areas of Radiology and Radiography but we will still need Radiographers to produce the Images and Radiologists to look at the ones that AI cannot read or flags up as abnormal”{28}.

The third was identified in response to our guidance content concerning HCPs on using “*AI for purpose it has been designed*”, where respondents recognised that training data, configuration, or environment will change (drift) over time. Subsequently, PEG-AI must direct HCPs to both understand and factor for drift when deciding whether AI use is appropriate for each patient.

These three points of guidance content will be added to this project’s next iteration.

### 5.4 Validity: lessons learned enabling future survey improvements

This pilot tested our survey for content and face validity. Were our questions sensible/appropriate/relevant? Did they cover what we intend them to cover? (Connell et al 2018). Overall, the subject matter of the survey was well received: the participants were interested in and engaged with the idea of AI being used in healthcare in the future and generously shared their views. Broadly speaking, our questions were pitched correctly to achieve our aims, but reviewing participants’ responses has given us insight on how we could improve the survey instrument prior to deployment in a future larger study.

On a practical front, participants noted that the survey was long and likely fatigued respondents. Whilst these comments were made by respondents who nonetheless completed the survey, it is quite possible that the length will have put potential respondents off. A small number of participants submitted their responses without fully completing the survey, and many participants’ responses went from being detailed at the start to being much briefer at the end. All this suggests that the instrument requires shortening to maximise the number and quality of responses.

There was also a view expressed that a few questions were confusing for those who were ‘not academic’. In our view, this is less likely to do with not being academic per se and rather familiarity with the material, but speaks to the importance of trying to simply all the language further. Of help will likely be not using any contractions (such as HCP) which we had explained on first use but which some participants who reported they were confusing.

We found that we had many responses that were not focussed on HCP use of AI, and instead focussed on concerns about AI or healthcare more generally. Apparently anticipating this, one respondent suggested that questions could encourage participants to explore different categories and use-cases of AI, and that examples could be used to illustrate scenarios to elicit answers.

Finally, we were told by one respondent that our question about protected characteristics made little sense:

“For example, everyone is protected by the “age” characteristic because everyone has an age and you can’t be discriminated against because of this. This is the same for religion/belief because you also can’t be discriminated against due to lack of belief. I assumed you were asking more so about marginalised groups, and answered accordingly.” {11}

This survey’s next iteration will aim to ask fewer and more concise questions using simpler language. It will, however, embrace longform throughout and avoid contractions. Our questions will more directly focus participants on how AI is used by HCPs and utilise short vignettes to aid participants’ visualisation. Finally, we will invite participants to tell us about their characteristics, protected or not, and if (or how) those characteristics could (or have) put them at risk of experiencing inequality arising from AI use. We think that this last point is especially important as it contextualises the risks that marginalised groups can be exposed to, and identifying and reporting those risks is essential to preventing them in the future.

## 6 Discussion

Much has been written regarding the need for trust from those who would use AI (Sheir et al., 2024), but we must ensure that AI meets the needs of people, and not the other way round (Pfeifer-Chomiczewska 2023). Society looks to regulatory mechanisms to achieve this by requiring that identifiable AI risks are mitigated (Davis, & Birtwistle 2023). Ideally, responsibilities that ought to be held by interested actors should be proactively determined and a legal framework built to reflect those allocated obligations (Berber & Srećković, 2024; Davis, & Birtwistle 2023, Smith et al., 2023b). Internationally, regulatory practices are still maturing in this area; some jurisdictions regulate AI as a whole, others determine regulations sector-by-sector (Finocchiaro 2024). Within the UK, there is a noted gap in regulation for AI use in healthcare, which is surprising given that its adoption will impact directly upon humans who are at their most vulnerable (Smith 2021).

This study was the starting point for a body of work that aims to generate cross-professional ethical guidance for AI use for health care professionals. Our pilot data, presented above, gives us an anchor for beginning wider consultation by (1) providing a small evidence base on which to begin building guidance content and (2) helping us refine and improve our survey tool for future and wider deployment.

Broadly, what we found was unsurprising, insofar as respondents supported all items of PEG-AI that championed harm avoidance, thereby promoting patient safety as a core ethical principle. This reflects broader discussions, for example, around trustworthiness, responsibility, and accountability in which safety is a core feature in AI adoption (Yazdanpanah et al. 2023). This emphasises the ethical imperative of doing good and avoiding harm; indeed, the entire enterprise of HCP regulation is to protect the public (Professional Standards Authority 2018). The mechanisms already in place which support this legitimate aim, such as record keeping, information governance, and fault reporting were highly popular—as were anti-maleficence suggestions, e.g., of removing the risk altogether by taking the AI out of service if the risk of harm was unmitigable. However, we might reach the point where the risk of using an AI might be lower than if the same task were solely performed by humans. In this instance, we will need to accept AI risk as the lesser of two evils and develop strategies that account for negative outcomes (Raper 2024).

There were, however, nuanced views in several areas, reflecting complexity and uncertainty, with a clear awareness of the impossibility of having AI that is perfect (just as we do not have perfect HCPs). Awkward problems were raised, such as the nature of bias in both healthcare and AI that cannot be removed, or that HCPs face having to compensate for AI imperfection whilst not necessarily being able to recognise problems that could present in AI recommendations. The concerns regarding bias and datasets reflect wider discourses of bias in AI, for example the presence of inadvertent historic biases affecting diversity, non-discrimination, and fairness when aiming to achieve trustworthy AI that can adhere to the principle of fairness (High-Level Expert Group on Artificial Intelligence 2019). Whilst we can insist on oversight of systems that are diverse in terms of background, culture, and disciplines (High-Level Expert Group on Artificial Intelligence 2019), bias will remain as an ongoing challenge which users must monitor for. The respondents noted the complex challenges of what it means to hold responsibility for using an AI when they are not the only actor; yet we can predict challenges in sharing that responsibility (e.g. with the person who commissioned the AI) (Smith et al., 2023b) in the absence of definitive policy prescribing how that responsibility will be held.

Respondents gave us pause for thought when considering whether patients ought to be specifically informed of, and consent to, AI use in their care. Transparency and honesty are critical in healthcare, but AI use might eventually become so ubiquitous and expected that it would be practically impossible to decline its use. Yet, this underlines the importance of continual review of PEG-AI to ensure that it keeps pace with healthcare practice and the expectations of those who are affected by its use. Although respondents highlighted the importance of transparency, the value of transparency in relation to AI is potentially ambiguous. Onora O’Neill (2002) noted how complete openness isn’t the best way to build trust as information can be manipulated to suit the aims of those reporting it. Instead “well-placed trust grows out of active inquiry rather than blind acceptance” (O’Neill, 2002). To achieve this, we must have HCPs who are trained to understand and interpret that data that is made transparent. Transparency without understandability does not really help in any way.

Educating HCPs will be paramount to AI’s safe adoption at the bedside. But this training *must* be fortified with authoritative policy dictating the standard of AI-informed practice. Its absence serves only to force HCPs to do the best they can with the non-AI-specific guidance that already exists, and risking their professional registrations should they misjudge.

It is important to note that this study is not an exercise in ethics by opinion poll (Salloch et al. 2014) or a post-hoc rationalisation of PEG-AI content that we have chosen. Rather, this is a starting point for discussion regarding how we can support healthcare professionals when they use AI in the future. Indeed, this pilot alone is insufficient to base future PEG-AI upon; rather, substantial further engagement is needed with all groups that would be affected by AI use. This would necessitate involvement of parties such as the healthcare regulators who would oversee the HCPs using AI to ensure that the final PEG-AI recommendations would complement the codes of conduct already in force (Ooi 2024), and patient groups to ensure that their rights are upheld (Cordovano et al 2024). Additionally, PEG-AI is not a fix-all; as our respondents pointed out, education to support HCPs for AI use in practice is vital. First steps are being made in this direction by Health Education England (2023), but it will be challenging to develop practice in an area that is lacking an evidence base.

The Federation of Medical Regulatory Authorities of Canada’s (FMRAC, 2022) Working Group on Artificial Intelligence and the Practice of Medicine want evidence garnered from real-world experiences to inform guidance or minimum expectations of their physicians. We agree that premature guidance development risks advising HCPs to be, for example, more-or-less risk-averse than is necessary; however, it has also been substantially argued that HCPs cannot be expected to employ AI in their practices in a vacuum of authoritative council (Smith et al., 2023a). The advantage of PEG-AI over the healthcare regulators’ codes of conduct is that whichever working group eventually leads it could dynamically update it in response to real-world experiences as the practice of AI develops, rather than having to re-specify fundamental standards required in healthcare practice. Once PEG-AI has iteratively improved over time, healthcare regulators may then more concretely decide if/how its principles could be rolled into codes of conduct.

**Fig. 1:**
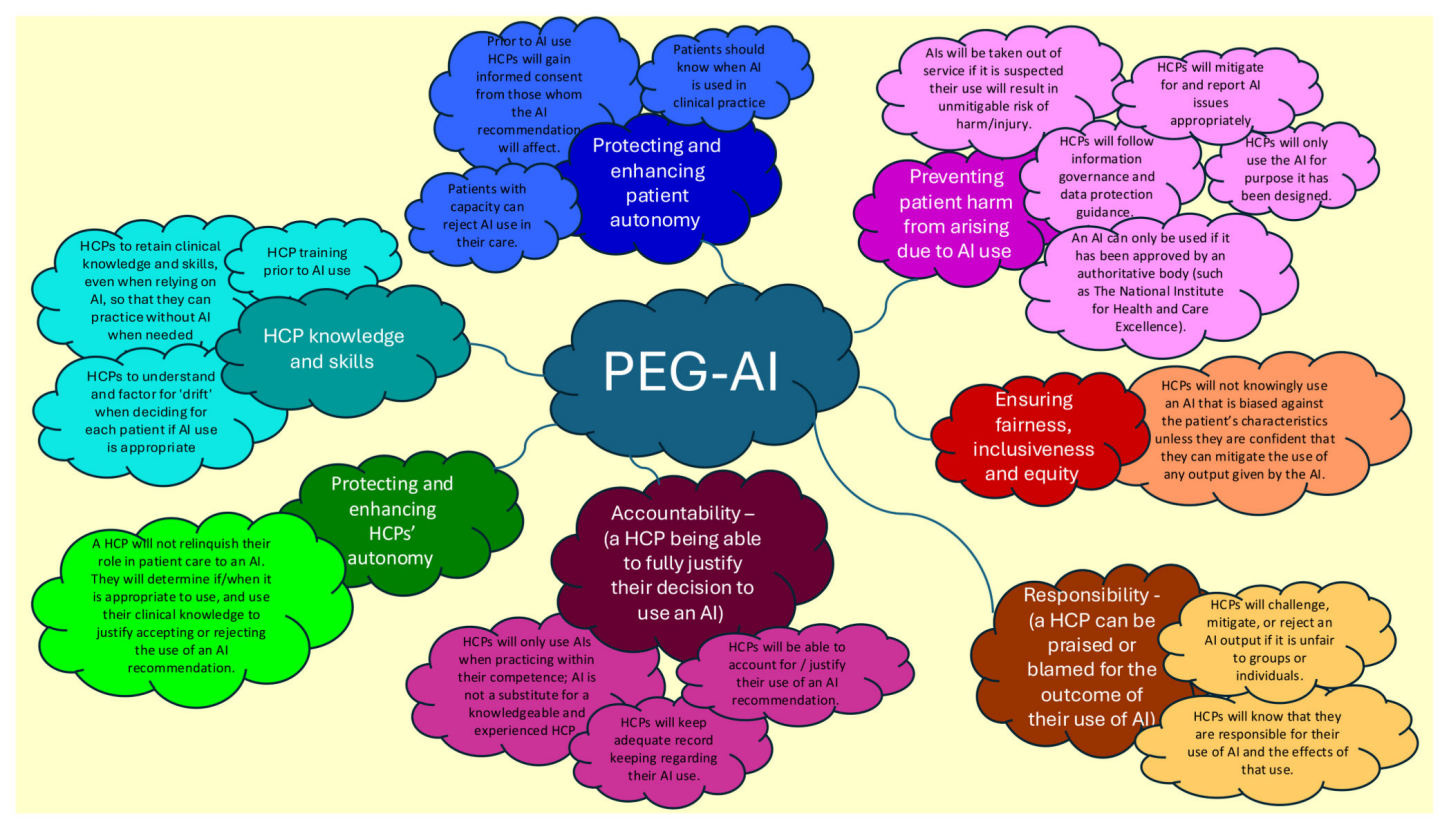
How the themes and guidance content could fit together

In the meantime, Fig. 1 offers a visual representation of how the themes and guidance content could fit together, demonstrating a provisional landscape of PEG-AI that might be considered.

However, the above illustration assumes that all themes and guidance content are used. Before taking this work to the next iteration beyond this pilot work, we must next consider how some themes or guidance can be revised, for example by combining the guidance of “Patients should know when AI is used in clinical practice” and “Prior to AI use HCPs will gain informed consent from those whom the AI recommendation will affect” as they are sufficiently closely linked to be managed together.

## 7 Limitations

We aimed to attract participants from diverse populations, representative of the UK population in terms of characteristics such as age, gender, socio-economic background, disability; however, the recruitment period was short (1 month) and our sample size relatively small. This meant that the results of this survey could only be generalised to represent the responder profile.

Our social media recruitment strategy allowed us to use limited resources to attract participants, however, we acknowledge the resulting selection bias due to the exclusion of wider populations who are not active on social media.

## 8 Conclusion

In this project, our exploratory pilot study gathered and analysed cross-sectional attitudinal and qualitative data from 42 people regarding our proposed PEG-AI.

The attitudinal survey found that respondents generally agreed with the proposed PEG-AI with accompanying qualitative data that helped us to understand why they had given each attitudinal score. The data indicated three further items of content; that HCPs will need training prior to their using AI, that HCPs should retain knowledge and skills so that they can practice independently without AI when needed, and that PEG-AI must direct HCPs to both understand and factor for drift when deciding whether AI use is appropriate for each patient. These will be incorporated into the next iteration of this work.

We recommend that this work is continued. Subject to further funding, we plan to take this study further to a wider study involving the next iteration of this survey, interviews with interested parties regarding PEG-AI, and an iterative Delphi process (comprising an initial co-creation workshop followed by iterative consensus building) to enable experts to reach consensus regarding recommendations for the content of PEG for AI use in healthcare. We aim for this work to inform both the Professional Standards Authority and the ten healthcare regulators that they oversee (Professional Standards Authority, 2024) as they develop regulatory strategies in this area. We would be delighted to hear from all who would be interested in developing this work with us.

## Figures and Tables

**Table 1:** Participants quantitative responses to guidance themes

Proposed themes	Essential	Important	Somewhat important	Not important	Don’t know
Preventing patient harm from arising due to AI use	39	2	0	1	0
Ensuring fairness, inclusiveness and equity	27	12	3	0	0
Protecting and enhancing patient autonomy	19	16	5	0	2
Protecting and enhancing HCPs’ autonomy	15	12	8	0	7
Accountability – (a HCP being able to fully justify their decision to use an AI)	23	14	1	1	3
Responsibility—(a HCP can be praised or blamed for the outcome of their use of AI)	16	12	5	3	6

**Table 2:** Participants quantitative responses to guidance content via the 5-point ordinal scale

Proposed guidance content	Essential	Important	Somewhat important	Not important	Don’t know	No response
An AI can only be used if it has been approved by an authoritative body (such as The National Institute for Health and Care Excellence)	26	11	2	1	2	0
HCPs will only use the AI for purpose it has been designed	28	8	2	1	2	1
HCPs will follow information governance and data protection guidance. E.g. Information governance and data protection https://www.england.nhs.uk/long-read/information-governance-and-data-protection/	33	7	1	0	0	1
HCPs will mitigate for and report AI issues appropriately (e.g. Medi-cines and Healthcare products Regulatory Agency’s Yellow Card Scheme https://yellowcard.mhra.gov.uk/) when they arise (for example, suspected error, suspected bias etc.)	32	8	1	0	0	1
AIs will be taken out of service if it is suspected their use will result in unmitigable risk of harm/injury	38	2	0	0	1	1
HCPs will not knowingly use an AI that is biased against the patient’s characteristics unless they are confident that they can mitigate the use of any output given by the AI	23	7	3	0	8	1
HCPs will challenge, mitigate, or reject an AI output if it is unfair to groups or individuals	29	7	3	0	2	1
Patients should know when AI is used in clinical practice	23	9	7	1	1	1
Prior to AI use HCPs will gain informed consent from those whom the AI recommendation will affect	20	6	6	5	4	1
Patients with capacity can reject AI use in their care	18	9	6	2	5	2
A HCP will not relinquish their role in patient care to an AI. They will determine if/when it is appropriate to use, and use their clinical knowl-edge to justify accepting or rejecting the use of an AI recommendation	28	9	1	0	2	2
HCPs will only use AIs when practicing within their competence; AI is not a substitute for a knowledgeable and experienced HCP	34	4	0	0	2	2
HCPs will be able to account for / justify their use of an AI recommen-dation	25	12	1	0	2	2
HCPs will keep adequate record keeping regarding their AI use	28	6	3	1	1	3
HCPs will know that they are responsible for their use of AI and the effects of that use	26	10	0	0	3	3

**Table 3:** Aggregated responder demographics

Age ranges:	Ethnicity:
18–29—5 30–39—7 40–49—4 50–59—13 60–69—5 70–79—2 80–89—5 90–99—1 Total = 42	Ethnicity: Black—1 White/Asian mixed - 1 Chinese—2 Nigerian—1 White British—21 White non-British—1 White Scottish—2 White English—2 English/British—1 White – 7 White other—1 N/A—1 No answer—1 Total = 42
Gender:	Sexual orientation:
Female—30 Male—11 No answer—1 Total = 42	Heterosexual/straight—31 Lesbian—2 Pansexual—1 Bisexual—1 Prefer not to say / Declined to answer—7 Total = 42
Participants’ interest in AI:	
Patient—19 Healthcare professional (HCP)—13	
○ *Registered with which regulator?:* ▪ General Medical Council—8 ▪ Nursing and Midwifery Council—4 ▪ HCPC—1	
Academic with an interest in PEG-AI for healthcare —7 Working for the Professional Standards Authority or one of the 10 regulators overseen by the Professional Standards Authority for Health and Social Care—1 An otherwise interested person—3 Total = 43	
*One participant indicated that they are both a registered healthcare professional and an academic, so the total here is greater than 42*	
Protected characteristic(s) as per the Equality Act 2010:	
Age—8 Disability—18 Gender reassignment—2 Marriage and civil partnership—5 Pregnancy and maternity—5 Race—6 Religion or belief—6 Sex—9 Sexual orientation—6	
*These demographic data total more than 42 as some participants identified more than one characteristic*	
Characteristic(s) which are not covered by the Equality Act 2010 which puts them at risk of or experiencing inequality:	
Pensioner—1 Single parent—1 Low income/poverty—3 Unpaid carer—2 Neurodivergent, but does not relate to having a disability—1 Experience of homelessness—1 Left education early—1 Fertility treatment access due to same sex marriage—1 Widowed—1	

## Data Availability

We have published a de-identified version of the survey data as Open Data in University of Bristol’s Research Data Repository. This is available at: Smith, H., Ives, J. (2024). Professional Ethical Guidance for Healthcare AI Use (PEG-AI). https://doi.org/10.5523/bris.2uj7vq3dbhbcz2kh8n5t91txh3.

## Declarations

1 We recognise that this is an unusually long commentary regarding responder demographics for this kind of paper. We felt that this level of discussion was needed as it is important that readers are aware of and conscientiously consider the voices of those who are at risk of the negative effects of AI adoption.

2 We appreciate that NICE does not regulate medical technology. However, we used NICE as an example of an authoritative body that all participants are likely to be familiar with. The MHRA may have been more apposite, but less well known to the wider public.

## References

[R1] Berber A, Srećković S (2024). When something goes wrong: Who is responsible for errors in ML decision-making?. AI & Socciety.

[R2] Central digital & data office (2020). data ethics framework.

[R3] Connell J, Carlton J, Grundy A, Taylor Buck E, Keetharuth AD, Ricketts T, Barkham M, Robotham D, Rose D, Brazier J (2018). The importance of content and face validity in instrument development: lessons learnt from service users when developing the recovering quality of life measure (ReQoL). Qual Life Res.

[R4] Connor L, Dean J, McNett M, Tydings DM, Shrout A, Gorsuch PF, Hole A, Moore L, Brown R, Melnyk BM, Gallagher-Ford L (2023). Evidence-based practice improves patient outcomes and healthcare system return on investment: findings from a scoping review. Worldview Evid Based Nurs.

[R5] Cordovano G, deBronkart D, Downing A, Duron Y, Glenn LK, Holdren J, Karmo M, Lewis D, Murphy M, Robinson V, Salmi L (2024). AI Rights For Patients: written by patients experts and community leaders. The Light Collective.

[R6] Davis M, Birtwistle M (2023). Regulating AI in the UK. Ada Lovelace Institute.

[R7] Department for Science, Innovation and Technology, Department of Health and Social Care (2023). Press release: New £100 million fund to capitalise on AI’s game-changing potential in life sciences and healthcare.

[R8] Department of Health and Social Care (2021). A guide to good practice for digital and data-driven health technologies.

[R9] Equality Act 2010 c.15.

[R10] Federation of Medical Regulatory Authorities of Canada (2022). Summary statement on artificial intelligence and the practice of medicine.

[R11] Finocchiaro G (2024). The regulation of artificial intelligence. AI & Soc.

[R12] Floridi L, Strait A (2020). Ethical foresight analysis: what it is and why it is needed?. Mind Mach.

[R13] General Medical Council (2024). Good medical practice: general medical council.

[R14] Health and Care Professions Council (2016). Standards of conduct, performance and ethics.

[R15] Health Education England (2023). AI and Digital Healthcare Technologies Capability framework.

[R16] High-Level Expert Group on Artificial Intelligence (2019). Ethics Guidelines for Trustworthy AI.

[R17] Hsieh H-F, Shannon SE (2005). Three Approaches to Qualitative Content Analysis. Qual Health Res.

[R18] Munroe R Standards XCKD.

[R19] NHS AI Lab & Health Education England (2022). Understanding healthcare workers’ confidence in AI. Report 1.

[R20] NHSX (2020). A Buyer’s Guide to AI in Health and Care.

[R21] NMC (2018). The code for nurses and midwives.

[R22] Obermeyer (2019). Dissecting Racial Bias in an Algorithm Used to Manage the Health of Populations.

[R23] O’Neill O (2002). Reith Lectures 2002: A Question of Trust. BBC.

[R24] Ooi K (2024). Using Artificial Intelligence in Patient Care—Some Considerations for Doctors and Medical Regulators. ABR.

[R25] Pfeifer-Chomiczewska K (2023). Intelligent service robots for elderly or disabled people and human dignity: legal point of view. AI & Soc.

[R26] Professional Standards Authority (2018). Professional healthcare regulation in the UK explained.

[R27] Professional Standards Authority for Health and Social Care (2024). Find a regulator.

[R28] Raper R (2024). A comment on the pursuit to align AI: we do not need value-aligned AI, we need AI that is risk-averse. AI & Soc.

[R29] Ruskin KJ, Corvin C, Rice SC (2020). Autopilots in the operating room: safe use of automated medical technology. Anesthesiology.

[R30] Salloch S, Vollmann J, Schildmann J (2014). Ethics by opinion poll? The Functions of Attitudes Research for Normative Deliberations in Medical Ethics Journal of Medical Ethics.

[R31] Secretary of State for Science, Innovation and Technology (2024). A pro-innovation approach to AI regulation: government response to consultation.

[R32] Sjoding (2020). Racial Bias in Pulse Oximetry Measurement.

[R33] Smith H (2021). Clinical AI: opacity, accountability, responsibility and liability. AI & Soc.

[R34] Smith H, Manzini A, Ives J (2022). Inclusivity in TAS research: An example of EDI as RRI. Journal of Responsible Technology.

[R35] Smith H, Downer J, Ives J (2023a). Clinicians and AI use: where is the professional guidance?. J Med Ethics.

[R36] Smith (2023b). Artificial intelligence in clinical decision-making: Rethinking personal moral responsibility. Bioethics.

[R37] Society of Radiographers (2021). Artificial Intelligence: Guidance for clinical imaging and therapeutic radiography professionals, a summary by the Society of Radiographers AI working group. Radiography.

[R38] Sullivan GM, Artino AR (2013). Analyzing and interpreting data from likert-type scales. J Grad Med Educ.

[R39] The Artificial Intelligence Act - Regulation (EU) 2024/1689.

[R40] Thimbleby H (2021). Fix IT: See and solve the problems of digital healthcare.

[R41] Trendall (2019). Gender bias concerns raised over GP app.

[R42] UK Parliament (2023). UK disability statistics: Prevalence and life experiences.

[R43] UNESCO (2021). Recommendation on the Ethics of Artificial Intelligence.

[R44] World Health Organization (2021). Ethics and governance of artificial intelligence for health.

[R45] Yazdanpanah V, Gerding EH, Stein S (2023). Reasoning about responsibility in autonomous systems: challenges and opportunities. AI & Soc.

